# Syntheses and crystal structures of bis­(2,3-di­methyl­pyrazine-κ*N*)di­iodido­cadmium(II) and *catena*-poly[[di­iodido­cadmium(II)]-μ-2,3-di­methyl­pyrazine-κ^2^*N*^1^:*N*^4^]

**DOI:** 10.1107/S2056989026002896

**Published:** 2026-03-24

**Authors:** Christian Näther

**Affiliations:** a Institut für Anorganische Chemie, Universität Kiel, Max-Eyth.-Str. 2, 24118 Kiel, Germany; Universität Greifswald, Germany

**Keywords:** crystal structure, discrete complex, coordination polymer, synthesis, cadmium iodide, 2,3-di­methyl­pyrazine

## Abstract

The reaction of cadmium iodide with 2,3-di­methyl­pyrazine leads to the formation of two compounds with the compositions [CdI_2_(C_6_H_8_N_2_)_2_] or [CdI_2_(2,3-di­methyl­pyrazine)_2_] (**1**) and [CdI_2_(C_6_H_8_N_2_)]_*n*_ or [CdI_2_(2,3-di­methyl­pyrazine)]_*n*_ (**2**). The asymmetric unit of **1** is built up of one Cd^II^ cation as well as two iodide anions and two 2,3-di­methyl­pyrazine ligands representing complete mol­ecules. The Cd^II^ cations are tetra­hedrally coordinated, forming discrete complexes that are connected *via* weak C—H⋯I inter­actions. The asymmetric unit of **2** consists of one Cd^II^ cation and two crystallographically independent iodide anions that are located on a crystallographic mirror plane, as well as one 2,3-di­methyl­pyrazine ligand that is located on a twofold rotation axis. The Cd^II^ cations are tetra­hedrally coordinated by two iodide anions and two 2,3-di­methyl­pyrazine ligands into corrugated chains. As in **1**, inter­molecular C—H⋯I inter­actions are observed. Comparison of the experimental powder patterns with those calculated from single-crystal data proves that pure compounds have been obtained. The crystal structures are compared with those of related *MX*_2_ (*M* = Zn, Cd, *X* = Cl, Br, I) coordination compounds with pyrazine and 2,3-di­methyl­pyrazine, that are reported in the literature.

## Abstract 2

The syntheses and crystal structures of [CdI_2_(2,3-di­methyl­pyrazine)_2_](**1**) and [CdI_2_(2,3-di­methyl­pyrazine)]_*n*_ (**2**) are reported. Compound **1** consists of discrete complexes, whereas in **2** the Cd cations are linked into chains by bridging 2,3-di­methyl­pyrazine ligands.

## 1. Chemical context

For many years we and others have been inter­ested in the synthesis, crystal structures and thermal properties of transition metal halide compounds with one- and twofold positively charged cations and N-donor coligands (Kromp & Sheldrick, 1999 ▸; Peng *et al.*, 2010 ▸; Li *et al.*, 2005 ▸; Näther & Jess, 2002 ▸). For one definite N-donor ligand and one definite halide anion, compounds with a different ratio between the metal halide and the coligand are usually observed. Many years ago, we found that many coligand-rich Cu^I^ compounds lose their coligands in different steps upon heating, leading to the formation of coligand-deficient phases as inter­mediates (Näther & Jess, 2001 ▸; Näther *et al.*, 2001 ▸, 2002 ▸). Later we also observed that some metal halide compounds with Zn^II^ or Cd^II^ show a similar thermal reactivity, even if it is not as pronounced as for the Cu^I^ compounds (Neumann *et al.*, 2018*a* ▸,*b* ▸). This can be traced back to the fact that in copper(I) compounds, a number of different Cu*X* substructures such as rings or layers are observed, which are not observed for Zn^II^ compounds and only rarely for Cd^II^ compounds. However, some examples exist where the metal cations in Zn*X*_2_ or Cd*X*_2_ compounds are linked by bridging halide anions into, for example, dinuclear units (Geringer *et al.*, 2020 ▸; Panda *et al.*, 2024 ▸; Rogers, 2020 ▸; Pickardt & Staub, 1997 ▸).
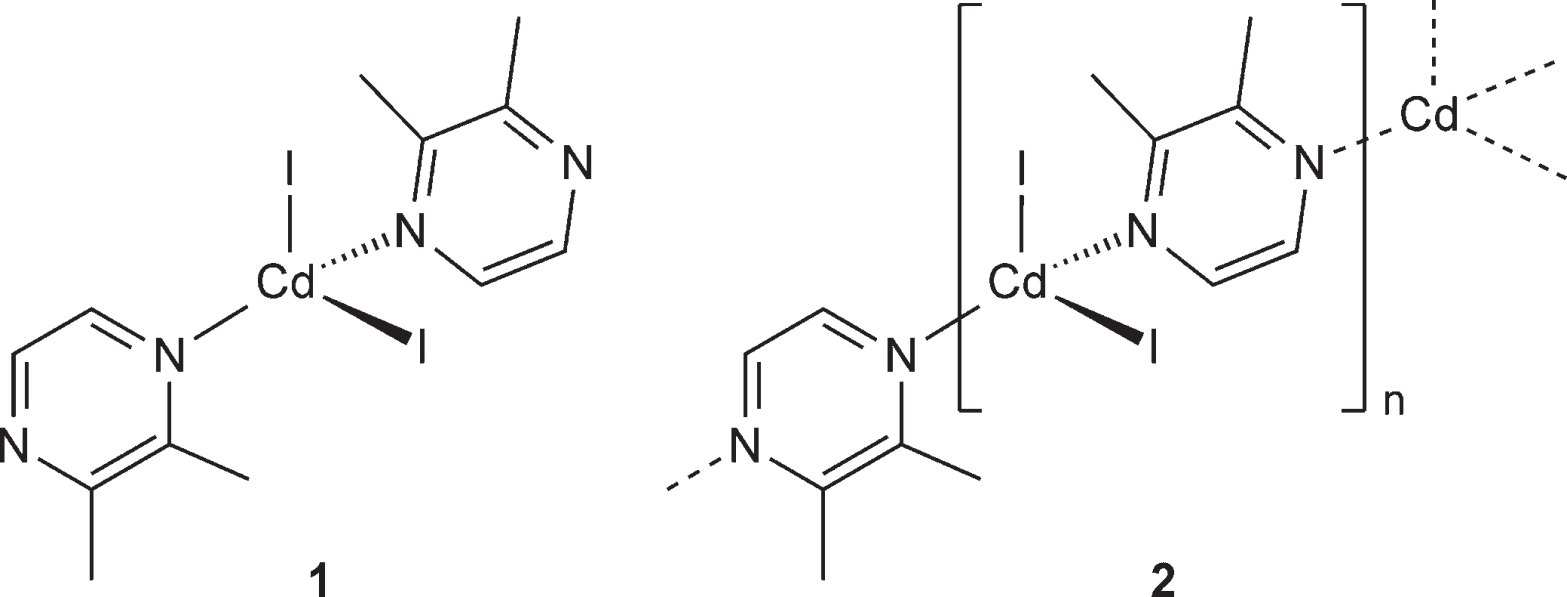


However, compounds with a different ratio between the metal halide and the N-donor ligands can also be obtained if bridging, instead of monocoordinating coligands, are used. This is the case, for example, for compounds with pyrazine as coligand, for which ligand-rich and ligand-deficient compounds with the composition Zn*X*_2_(pyrazine)_2_ [*X* = Cl (refcode REMPAB; Bhosekar *et al.*, 2006 ▸) and *X* = Br (EBOLAI; Bourne *et al.*, 2001 ▸)] and Zn*X*_2_(pyrazine) (*X* = Cl, Br, I) [*X* = Cl (refcode TISTAQ; Pickardt & Staub, 1997 ▸), *X* = Br (EBOKUB; Bourne *et al.*, 2001 ▸), *X* = I (ISOPOV; Song *et al.*, 2004 ▸ and ISOPOV01; Bhosekar *et al.*, 2006 ▸)] are reported. Surprisingly, with Cd^II^ only the pyrazine-deficient compounds with the composition Cd*X*_2_(pyrazine) are listed in the Cambridge Structural Database (CSD Version 5.43, 2025; Groom *et al.*, 2016 ▸) [*X* = Cl (refcode TISSUJ; Pickardt & Staub, 1996 ▸, TISSUJ01; Bailey & Pennington, 1997 ▸, Lusi *et al.*, 2011 ▸), *X* = Br (RINSIQ; Bailey & Pennington, 1997 ▸, RINSIQ01, Pickardt & Staub, 1996 ▸), *X* = I (RINSOW; Bailey & Pennington, 1997 ▸, Pickardt & Staub, 1997 ▸)].

In the course of our systematic project, we became inter­ested in Zn*X*_2_ and Cd*X*_2_ compounds with 2,3-di­methyl­pyrazine, in which the metal coordination is more difficult, because of the methyl groups that are adjacent to the coordinating N atom. With Zn*X*_2_ (*X* = Cl, Br, I) chloride-bearing compounds with the composition [ZnCl_2_(2,3-di­methyl­pyrazine)_2_] and [ZnCl_2_(2,3-di­methyl­pyrazine)]_*n*_ were characterized (Näther & Bhosekar, 2025*a* ▸). The former compound consists of discrete tetra­hedral complexes, whereas in the second compound the tetra­hedra are linked into chains *via* the 2,3-di­methyl­pyrazine ligands. [ZnBr_2_(2,3-Di­methyl­pyrazine)_2_] is isotypic with the corresponding ZnCl_2_ compound (Yang *et al.*, 2025 ▸) and in [ZnBr_2_(2,3-di­methyl­pyrazine)]_*n*_ the metal cations are linked into chains (Näther & Bhosekar, 2025*b* ▸). Finally, [ZnI_2_(2,3-di­methyl­pyrazine)] is also reported and is isotypic to its bromide analog (Näther & Bhosekar, 2026 ▸). Compounds based on Cd*X*_2_ (*X* = Cl, Br, I) are not reported and therefore, in our initial experiments we tried to prepare such compounds. Here we report on our investigations.

## 2. Structural commentary

**Figure 1:**
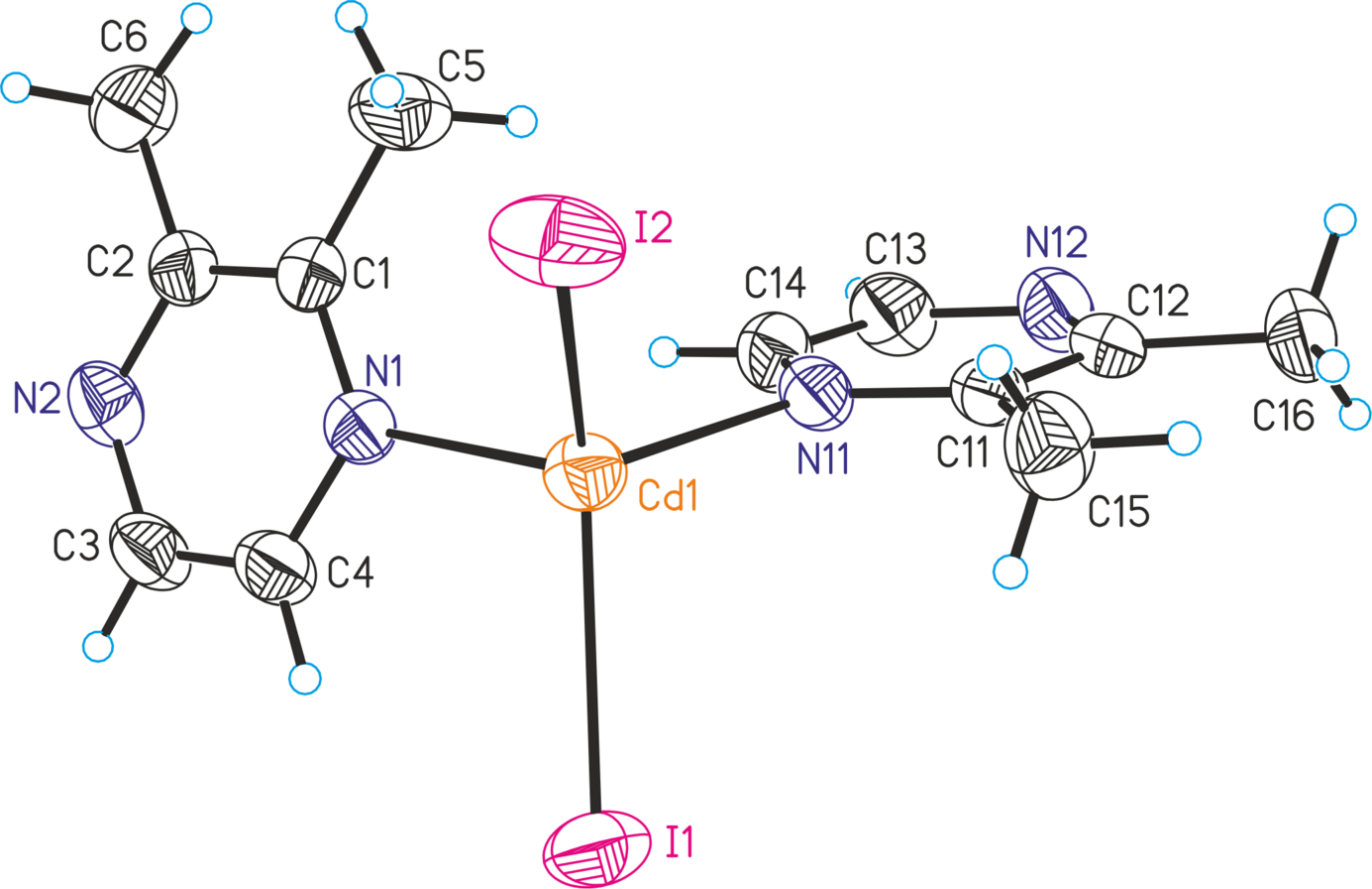
Crystal structure of **1** with labeling and displacement ellipsoids drawn at the 50% probability level.

The asymmetric unit of the 2,3-di­methyl­pyrazine-rich compound [CdI_2_(2,3-di­methyl­pyrazine)_2_] (**1**) consists of one Cd^II^ cation, as well as two crystallographically independent iodide anions and 2,3-di­methyl­pyrazine ligands (Fig. 1 ▸). In the crystal structure, the Cd^II^ cations are surrounded by two iodide anions and two 2,3-di­methyl­pyrazine ligands, forming discrete tetra­hedral complexes. The bond angles deviate from the ideal geometry, which shows that the tetra­hedra are strongly distorted (Table 1 ▸). The largest value of 120.373 (14) ° is observed for the I—Cd—I angle, which can be traced back to steric repulsion between the large halide anions.

It is noted that [ZnCl_2_(2,3-di­methyl­pyrazine)_2_] and [ZnBr_2_(2,3-di­methyl­pyrazine)_2_] also form discrete tetra­hedral complexes but they are not isotypic to **1** (Näther & Bhosekar, 2025*a* ▸,*b* ▸). It is also noted that a 2,3-di­methyl­pyrazine-rich compound with ZnI_2_ is unknown. However, similar compounds with pyrazine are reported. This includes [ZnCl_2_(pyrazine)_2_]_*n*_ (refcode REMPAB; Bhosekar *et al.*, 2006 ▸) and [ZnBr_2_(pyrazine)_2_]_*n*_ (EBOLAI; Bourne *et al.*, 2001 ▸ and EBOLAI01; Bhosekar *et al.*, 2006 ▸), in which the Zn^II^ cations are tetra­hedrally coordinated and linked into layers by the pyrazine ligands. ZnI_2_(pyrazine)_2_ as well as pyrazine-rich compounds with the composition Cd*X*_2_(pyrazine)_2_ (*X* = Cl, Br, I) are unknown. Finally, the reason why compound **1** as well as [ZnCl_2_(2,3-di­methyl­pyrazine)_2_] and [ZnBr_2_(2,3-di­methyl­pyrazine)_2_] form discrete complexes whereas the corresponding compounds with pyrazine form layers might originate from steric repulsion between the cation and the methyl group adjacent to the N atom, which makes a metal coordination more difficult.

**Figure 2:**
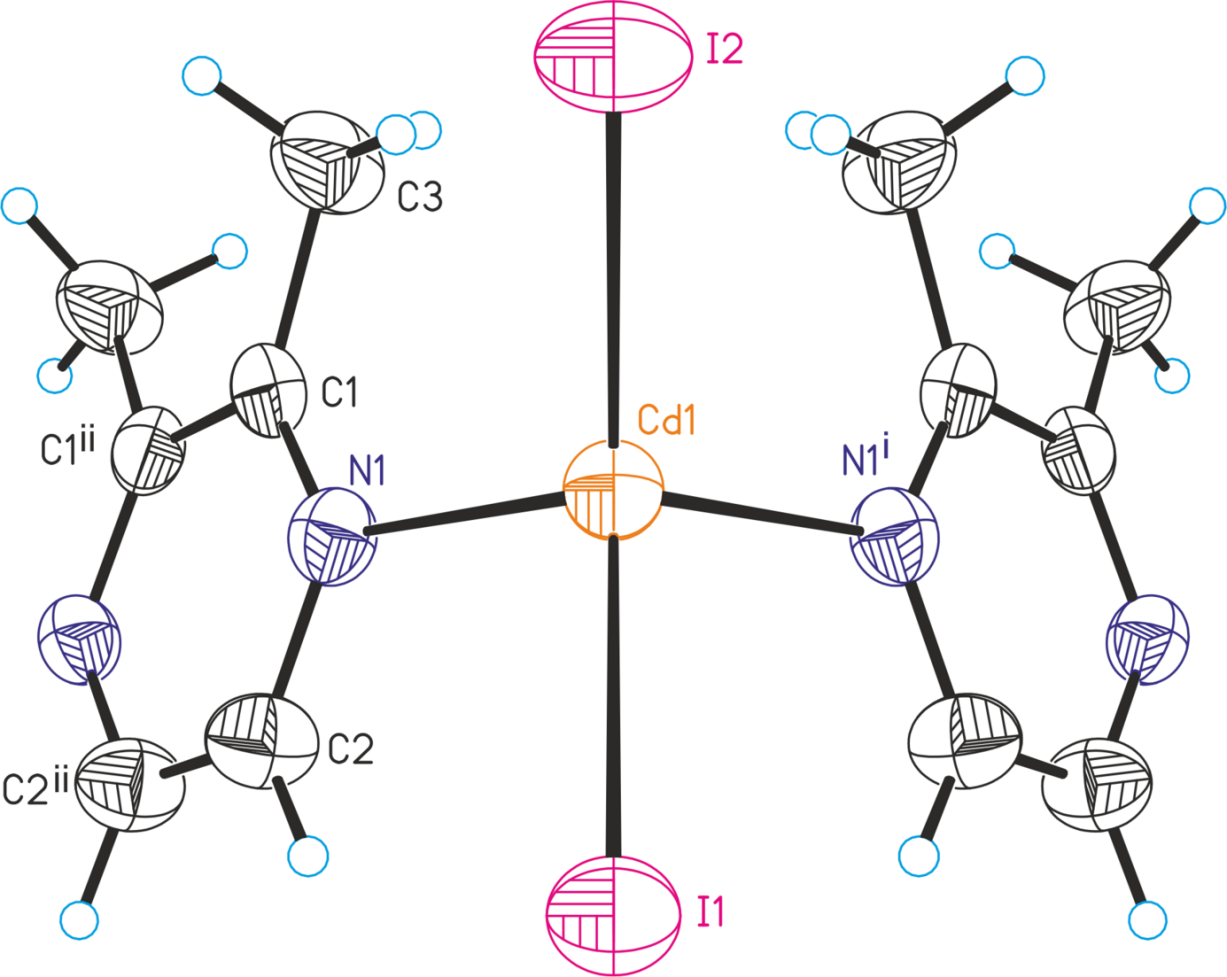
Crystal structure of **2** with labeling and displacement ellipsoids drawn at the 50% probability level. Symmetry codes for the generation of equivalent atoms: (i) *x*, *y*, −*z* + 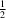; (ii) *x*, −*y* + 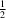, −*z* + 1.

**Figure 3:**
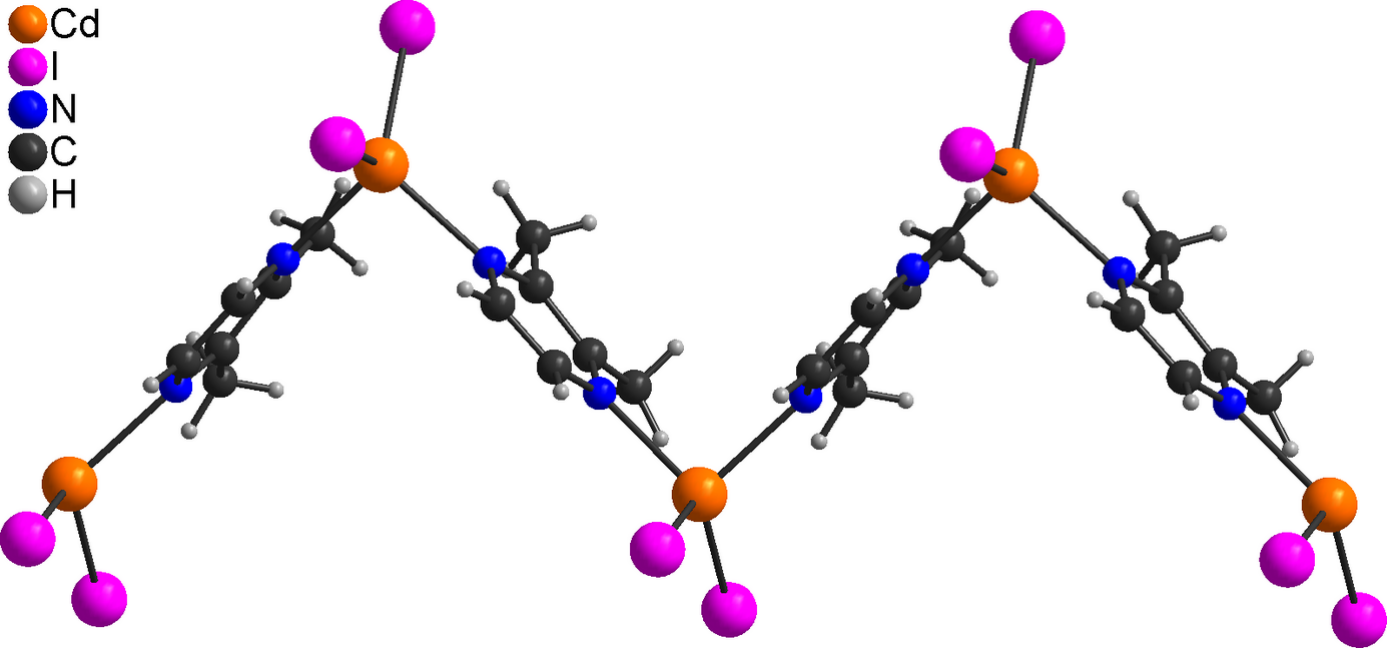
Crystal structure of **2** with view of a part of a chain.

The asymmetric unit of [CdI_2_(2,3-di­methyl­pyrazine)] (**2**) is built up of one Cd^II^ cation and two crystallographically independent iodide anions that are located on a crystallographic mirror plane, as well as one 2,3-di­methyl­pyrazine ligand that is located on a twofold rotation axis (Fig. 2 ▸). The Cd^II^ cations are fourfold coordinated by two iodide anions and two bridging 2,3-di­methyl­pyrazine ligands and are linked into corrugated chains by the coligands (Fig. 3 ▸). The bond angles deviate from the ideal values, which shows that the tetra­hedra are slightly distorted (Table 2 ▸). In contrast to **1**, the largest deviation is found for the N—Cd—N angles (Table 2 ▸).

Comparison of the structure of **2** with that of the isotypic Zn^II^ compounds ZnCl_2_(2,3-di­methyl­pyrazine) (Näther & Bhosekar, 2025*a* ▸) and ZnBr_2_(2,3-di­methyl­pyrazine) (Näther & Bhosekar, 2025*b* ▸) shows that neither is isotypic to compound **2**. In this context, it is noted that the corresponding compounds with pyrazine show different structures in which the Zn^II^ cations are linked into layers by the pyrazine ligands (REMPAB; Bhosekar *et al.*, 2006 ▸ and EBOLAI; Bourne *et al.*, 2001 ▸). This might also originate from the fact that the coordination to the N atom in the 2,3-di­methyl­pyrazine compounds is sterically hindered, which is not the case in the pyrazine compounds.

## 3. Supra­molecular features

**Figure 4:**
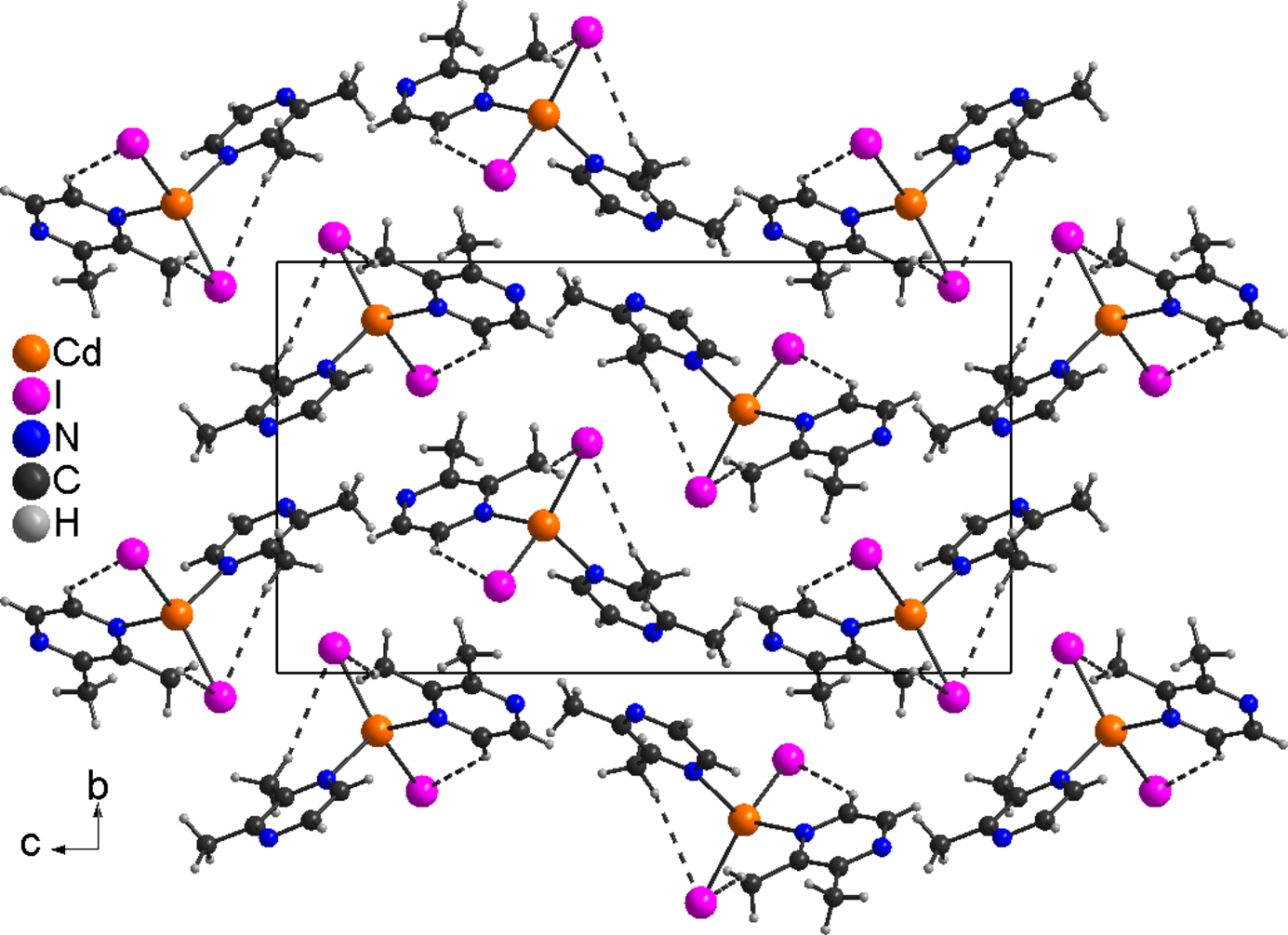
Crystal structure of **1** with intra­molecular C—H⋯I inter­actions shown as dashed lines.

In compound **1**, C—H⋯I inter­actions, especially between two of the methyl H atoms and both iodide anions, are observed within each complex (Fig. 4 ▸ and Table 3 ▸). The C—H⋯I angle is close to linear, indicating that this is a significant inter­action (Table 3 ▸). No such inter­actions are observed between the complexes.

**Figure 5:**
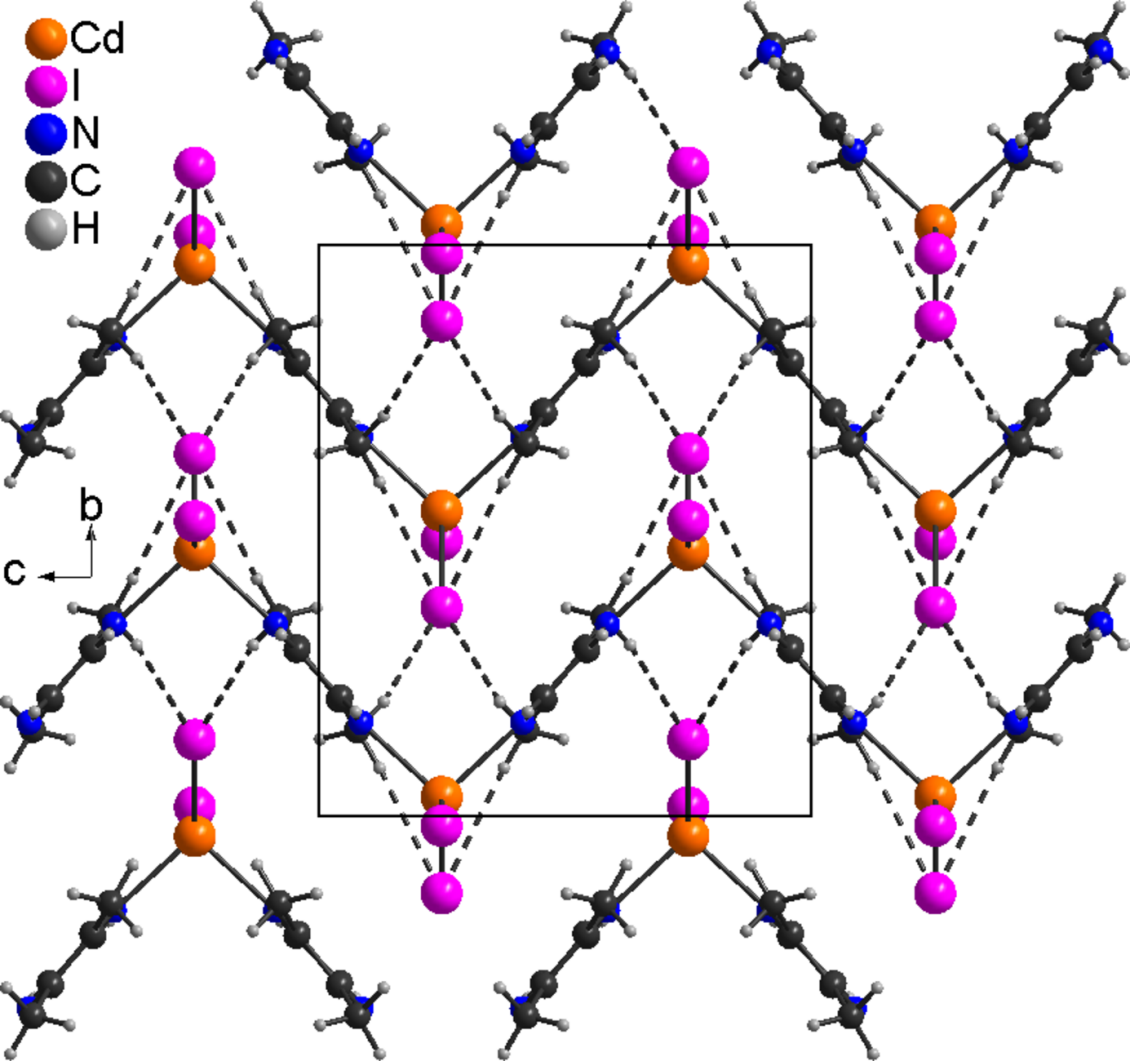
Crystal structure of **2** with inter­molecular C—H⋯I inter­actions shown as dashed lines.

In the 2,3-di­methyl­pyrazine-deficient compound **2**, the complexes are arranged into columns that elongate in the *c*-axis direction and are linked by inter­molecular C—H⋯I inter­actions (Table 4 ▸). The strongest C—H⋯I inter­actions are also observed between the methyl H atoms and the iodide anions (Fig. 5 ▸ and Table 4 ▸). In contrast to **1**, the H⋯I distances are shortened and both C—H⋯I angles are very close to linear, which indicate that these inter­actions are stronger than in **1**. These inter­actions lead to the formation of a three-dimensional network (Fig. 5 ▸).

## 4. Database survey

A search in the Cambridge Structural Database (CSD Version 5.43, 2025; Groom *et al.*, 2016 ▸) using CONQUEST (Bruno *et al.*, 2002 ▸) reveals that no coordination compounds with cadmium halides and 2,3-di­methyl­pyrazine as ligands are reported. However, some compounds with zinc halides are reported, as already mentioned in the *Chemical context* section. These include ZnCl_2_(2,3-di­methyl­pyrazine), ZnBr_2_(2,3-di­methyl­pyrazine) and ZnI_2_(2,3-di­methyl­pyrazine) (Näther & Bhosekar, 2025*a* ▸,*b* ▸, 2026 ▸), as well as ZnCl_2_(2,3-di­methyl­pyrazine)_2_ (Näther & Bhosekar, 2025*a* ▸) and ZnBr_2_(2,3-di­methyl­pyrazine)_2_ (Yang *et al.*, 2025 ▸). Finally, a compound with the composition [ZnI_2_(C_6_H_8_N_2_)(H_2_O)](H_2_O)_0.5_(C_6_H_8_N_2_)_0.5_ is also known (Näther & Bhosekar, 2026 ▸).

With the related ligand pyrazine, some compounds with CdI_2_ are also listed in the CSD. These include CdCl_2_(pyrazine) (refcode TISSUJ; Pickardt & Staub, 1996 ▸, TISSUJ01, Bailey & Pennington, 1997 ▸, Lusi *et al.*, 2011 ▸), CdBr_2_(pyrazine) (RINSIQ; Bailey & Pennington, 1997 ▸, RINSIQ01; Pickardt & Staub, 1996 ▸) and CdI_2_(pyrazine) (RINSOW; Bailey & Pennington, 1997 ▸; Pickardt & Staub, 1997 ▸).

## 5. Powder X-ray diffraction and thermoanalytical measurements

**Figure 6:**
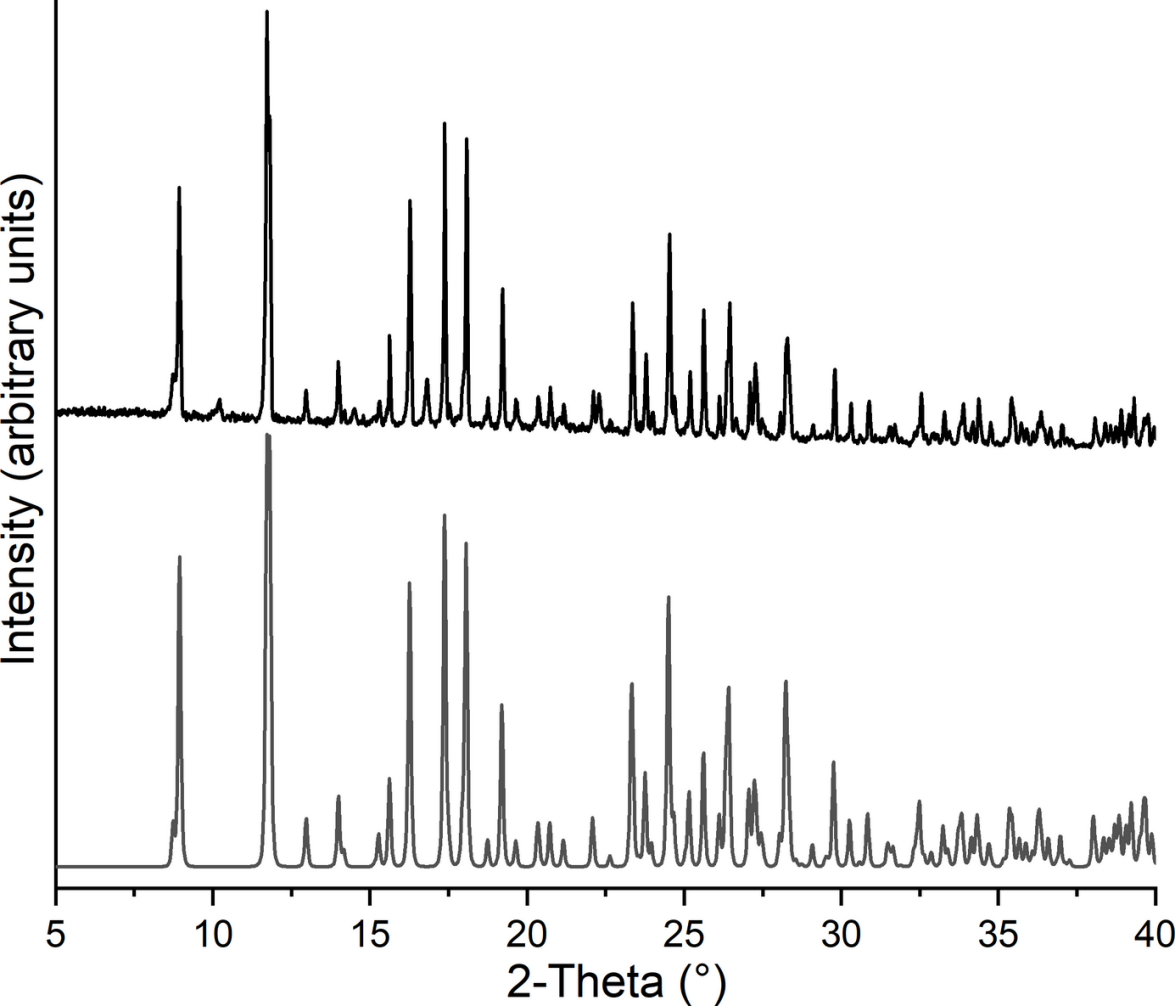
Experimental (top) and calculated (bottom) X-ray powder pattern of **1**.

Both compounds were additionally investigated by powder X-ray diffraction (PXRD). Comparison of the experimental patterns with those calculated from single crystal data proves, that pure phases were obtained (Figs. 6 ▸ and 7 ▸). In the pattern of compound **1** there is one peak of very low intensity at a Bragg angle of 10.2°, indicating traces of a second crystalline phase.

Measurements using thermogravimetry and differential thermoanalysis on compound **1** show that two mass losses are observed, which are accompanied by endothermic events in the DTA curve and are perfectly resolved as obvious from the DTG curve (Fig. S1). The experimental mass losses of 17.8 and 18.6% are in reasonable agreement with those calculated for the removal of each one 2,3-di­methyl­pyrazine ligand in each step (Δ*m*_calc._ = 18.6%). Therefore, it can be assumed that compound **2** has formed in the first mass loss and that the remaining ligands are emitted in the second mass loss.

TG-DTA measurements on **2** show only one mass loss at 471 K, which corresponds to the temperature where the second mass loss is observed for compound **1** (Fig. S2). The experimental mass loss of 22.8% is in perfect agreement with that calculated for the removal of one 2,3-di­methyl­pyrazine ligand (Δ*m*_calc._ = 22.8%).

## 6. Synthesis and crystallization

**General**

Cadmium iodide and 2,3-di­methyl­pyrazine were purchased from Sigma-Aldrich. The purity of both compounds was proven by powder X-ray diffraction.

**Synthesis of 1**

0.5 mmol (183.1 mg) of cadmium iodide and 1.0 mmol (108.1 mg) of 2,3-di­methyl­pyrazine were stirred in 4 mL of aceto­nitrile for 2 d. The precipitate was filtered off and dried. Single crystals were obtained using the same ratio of reactants without stirring.

**Synthesis of 2**

0.5 mmol (183.1 mg) of cadmium iodide and 0.5 mmol (54.1 mg) of 2,3-di­methyl­pyrazine were stirred in 3 mL of aceto­nitrile for 2 d. The precipitate was filtered off and dried. Single crystals were obtained using the same ratio of reactands without stirring.

**Experimental details**

The PXRD measurements were performed with a Stoe Transmission Powder Diffraction System (STADI P) with Cu *K*α_1_ radiation (λ = 1.540598 Å) equipped with a MYTHEN 1K detector and a Johansson-type Ge(111) monochromator.

The TG-DTA measurements were performed using a Linseis thermobalance in Al_2_O_3_ crucibles with 4°C/min in a flowing nitro­gen atmosphere.

## 7. Refinement

Crystal data, data collection and structure refinement details are summarized in Table 5 ▸. The C—H hydrogen atoms were positioned with idealized geometry (methyl H atoms allowed to rotate but not to tip) and were refined isotropically with *U*_iso_(H) = 1.2*U*_eq_(C) (1.5 for methyl H atoms).

## Supplementary Material

Crystal structure: contains datablock(s) 1, 2. DOI: 10.1107/S2056989026002896/yz2075sup1.cif

Structure factors: contains datablock(s) 1. DOI: 10.1107/S2056989026002896/yz20751sup2.hkl

Structure factors: contains datablock(s) 2. DOI: 10.1107/S2056989026002896/yz20752sup3.hkl

CCDC references: 2539546, 2539547

Additional supporting information:  crystallographic information; 3D view; checkCIF report

## Figures and Tables

**Figure 7:**
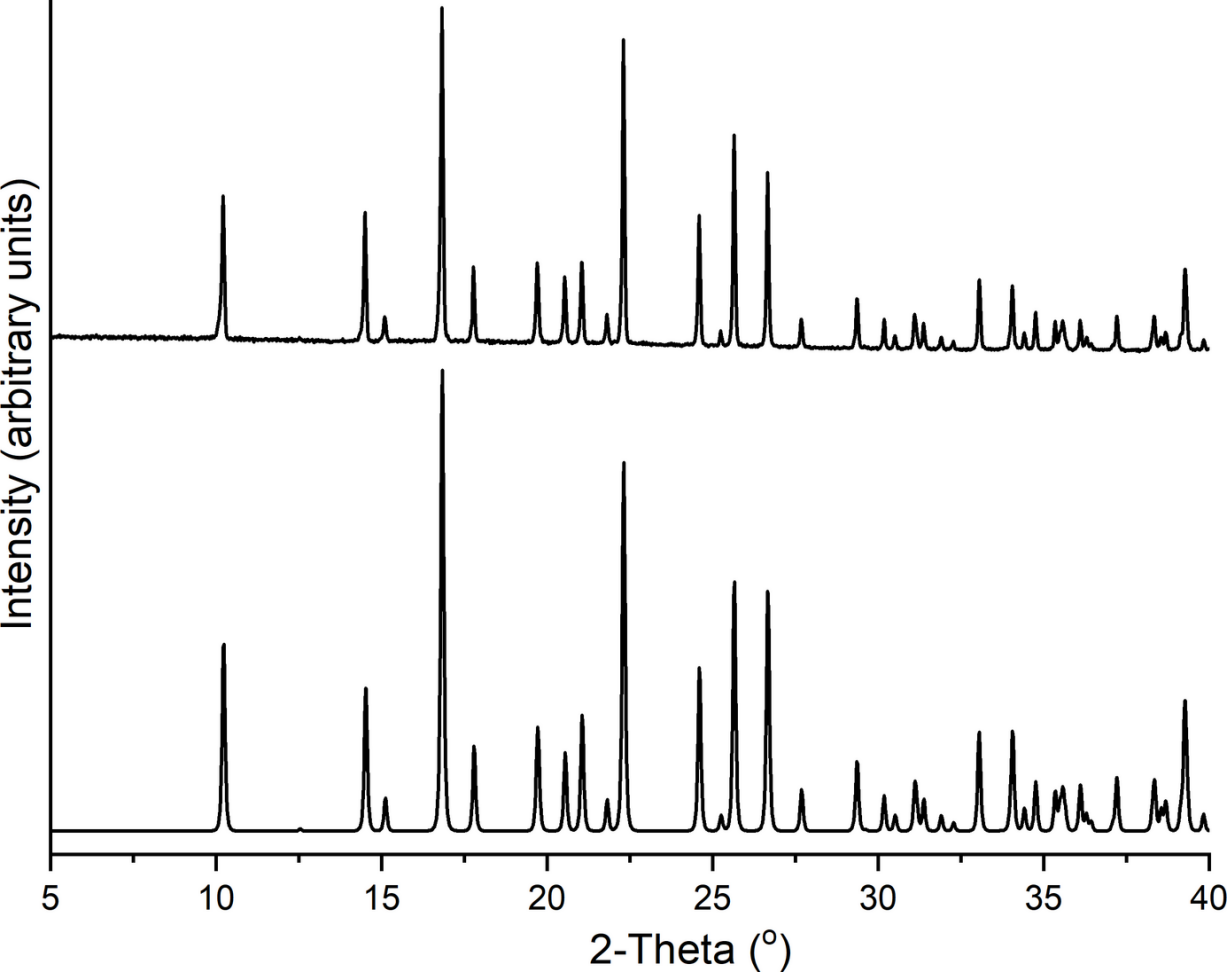
Experimental (top) and calculated (bottom) X-ray powder pattern of **2**.

**Table 1:** Selected geometric parameters (Å, °) for **1**[Chem scheme1]

Cd1—N1	2.329 (3)	Cd1—I2	2.7084 (4)
Cd1—N11	2.331 (3)	Cd1—I1	2.7309 (4)
			
N1—Cd1—N11	97.83 (10)	N1—Cd1—I1	102.85 (7)
N1—Cd1—I2	113.60 (7)	N11—Cd1—I1	106.28 (7)
N11—Cd1—I2	113.12 (7)	I2—Cd1—I1	120.373 (14)

**Table 2:** Selected geometric parameters (Å, °) for **2**[Chem scheme1]

Cd1—N1^i^	2.354 (3)	Cd1—I2	2.6941 (6)
Cd1—N1	2.354 (3)	Cd1—I1	2.7170 (7)
			
N1^i^—Cd1—N1	94.00 (17)	N1—Cd1—I1	105.18 (8)
N1—Cd1—I2	116.09 (8)	I2—Cd1—I1	117.27 (2)
N1^i^—Cd1—I1	105.18 (8)		

Symmetry code: (i) 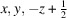.

**Table 3:** Hydrogen-bond geometry (Å, °) for **1**[Chem scheme1]

*D*—H⋯*A*	*D*—H	H⋯*A*	*D*⋯*A*	*D*—H⋯*A*
C4—H4⋯I1	0.93	3.13	3.848 (4)	136
C5—H5*A*⋯I2^i^	0.96	3.26	4.151 (5)	156
C15—H15*C*⋯I2	0.96	3.28	4.186 (5)	158

Symmetry code: (i) 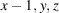.

**Table 4:** Hydrogen-bond geometry (Å, °) for **2**[Chem scheme1]

*D*—H⋯*A*	*D*—H	H⋯*A*	*D*⋯*A*	*D*—H⋯*A*
C2—H2⋯I1	0.93	3.19	3.915 (5)	136
C3—H3*A*⋯I2	0.96	3.08	4.036 (5)	175
C3—H3*C*⋯I2^ii^	0.96	3.14	4.081 (5)	167

Symmetry code: (ii) 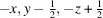.

**Table 5:** Experimental details

	**1**	**2**
Crystal data		
Chemical formula	[CdI_2_(C_6_H_8_N_2_)_2_]	[CdI_2_(C_6_H_8_N_2_)]
*M*_r_	582.49	474.34
Crystal system, space group	Monoclinic, *P*2_1_/*n*	Orthorhombic, *Pbcm*
Temperature (K)	293	293
*a*, *b*, *c* (Å)	7.6955 (4), 11.3271 (7), 20.4421 (13)	8.639 (1), 12.183 (1), 10.519 (1)
α, β, γ (°)	90, 98.112 (7), 90	90, 90, 90
*V* (Å^3^)	1764.06 (18)	1107.11 (19)
*Z*	4	4
Radiation type	Mo *K*α	Mo *K*α
μ (mm^−1^)	4.73	7.50
Crystal size (mm)	0.15 × 0.12 × 0.10	0.12 × 0.10 × 0.08

Data collection		
Diffractometer	Stoe *IPDS1*	Stoe Stadi-4
Absorption correction	Numerical (*X-RED* and *X-SHAPE*; Stoe, 2008 ▸)	ψ scan (*REDU*; Stoe 1990 ▸)
*T*_min_, *T*_max_	0.468, 0.549	0.300, 0.372
No. of measured, independent and observed [*I* > 2σ(*I*)] reflections	12802, 4166, 3277	1623, 1273, 1022
*R*_int_	0.034	0.018
(sin θ/λ)_max_ (Å^−1^)	0.660	0.639

Refinement		
*R*[*F*^2^ > 2σ(*F*^2^)], *wR*(*F*^2^), *S*	0.028, 0.071, 1.01	0.023, 0.056, 1.04
No. of reflections	4166	1273
No. of parameters	177	57
H-atom treatment	H-atom parameters constrained	H-atom parameters constrained
Δρ_max_, Δρ_min_ (e Å^−3^)	0.48, −0.68	0.65, −0.61

Computer programs: *X-AREA* (Stoe, 2008 ▸), *DIF4* and *REDU* (Stoe 1990 ▸), *SHELXT* (Sheldrick, 2015*a* ▸), *SHELXL* (Sheldrick, 2015*b* ▸), *DIAMOND* (Brandenburg, 1999 ▸), *XP* in *SHELXTL-PC* (Sheldrick, 2008 ▸) and *publCIF* (Westrip, 2010 ▸).

## supplementary crystallographic information

### Bis(2,3-dimethylpyrazine-κN)diiodidocadmium(II) (1) Crystal data

**Table:**

[CdI_2_(C_6_H_8_N_2_)_2_]	*F*(000) = 1080
*M**_r_* = 582.49	*D*_x_ = 2.193 Mg m^−^^3^
Monoclinic, *P*2_1_/*n*	Mo *K*α radiation, λ = 0.71073 Å
*a* = 7.6955 (4) Å	Cell parameters from 8000 reflections
*b* = 11.3271 (7) Å	θ = 11–27°
*c* = 20.4421 (13) Å	µ = 4.73 mm^−^^1^
β = 98.112 (7)°	*T* = 293 K
*V* = 1764.06 (18) Å^3^	Block, colorless
*Z* = 4	0.15 × 0.12 × 0.10 mm

### Bis(2,3-dimethylpyrazine-κN)diiodidocadmium(II) (1) Data collection

**Table:**

Stoe IPDS-1 diffractometer	3277 reflections with *I* > 2σ(*I*)
Phi scans	*R*_int_ = 0.034
Absorption correction: numerical (X-Red and X-Shape; Stoe, 2008)	θ_max_ = 28.0°, θ_min_ = 3.0°
*T*_min_ = 0.468, *T*_max_ = 0.549	*h* = −10→9
12802 measured reflections	*k* = −14→14
4166 independent reflections	*l* = −26→22

### Bis(2,3-dimethylpyrazine-κN)diiodidocadmium(II) (1) Refinement

**Table:**

Refinement on *F*^2^	Hydrogen site location: inferred from neighbouring sites
Least-squares matrix: full	H-atom parameters constrained
*R*[*F*^2^ > 2σ(*F*^2^)] = 0.028	*w* = 1/[σ^2^(*F*_o_^2^) + (0.0405*P*)^2^ + 0.5502*P*] where *P* = (*F*_o_^2^ + 2*F*_c_^2^)/3
*wR*(*F*^2^) = 0.071	(Δ/σ)_max_ = 0.002
*S* = 1.01	Δρ_max_ = 0.48 e Å^−^^3^
4166 reflections	Δρ_min_ = −0.68 e Å^−^^3^
177 parameters	Extinction correction: SHELXL-2016/6 (Sheldrick 2015b), Fc^*^=kFc[1+0.001xFc^2^λ^3^/sin(2θ)]^-1/4^
0 restraints	Extinction coefficient: 0.0063 (3)
Primary atom site location: dual	

### Bis(2,3-dimethylpyrazine-κN)diiodidocadmium(II) (1) Special details

**Table:**

Geometry. All esds (except the esd in the dihedral angle between two l.s. planes) are estimated using the full covariance matrix. The cell esds are taken into account individually in the estimation of esds in distances, angles and torsion angles; correlations between esds in cell parameters are only used when they are defined by crystal symmetry. An approximate (isotropic) treatment of cell esds is used for estimating esds involving l.s. planes.

### Bis(2,3-dimethylpyrazine-κN)diiodidocadmium(II) (1) Fractional atomic coordinates and isotropic or equivalent isotropic displacement parameters (Å^2^)

**Table:**

	*x*	*y*	*z*	*U*_iso_*/*U*_eq_	
Cd1	0.66284 (4)	0.35851 (2)	0.63661 (2)	0.03811 (9)	
I1	0.91725 (4)	0.21125 (2)	0.69702 (2)	0.05213 (10)	
I2	0.75133 (4)	0.55962 (3)	0.57769 (2)	0.06045 (12)	
N1	0.4860 (4)	0.3899 (2)	0.71854 (14)	0.0341 (6)	
C1	0.3409 (5)	0.4564 (3)	0.71153 (18)	0.0349 (7)	
C2	0.2466 (5)	0.4720 (3)	0.76515 (18)	0.0374 (8)	
N2	0.2996 (4)	0.4245 (3)	0.82422 (16)	0.0445 (7)	
C3	0.4466 (5)	0.3601 (4)	0.83032 (19)	0.0456 (9)	
H3	0.487150	0.325882	0.870983	0.055*	
C4	0.5395 (5)	0.3427 (3)	0.77855 (19)	0.0420 (8)	
H4	0.641429	0.297510	0.784996	0.050*	
C5	0.2810 (6)	0.5132 (5)	0.6460 (2)	0.0582 (12)	
H5A	0.158870	0.496394	0.632675	0.087*	
H5B	0.348121	0.482539	0.613616	0.087*	
H5C	0.297712	0.597047	0.649698	0.087*	
C6	0.0828 (6)	0.5459 (4)	0.7586 (2)	0.0560 (11)	
H6A	−0.002863	0.513566	0.724627	0.084*	
H6B	0.110167	0.625297	0.747174	0.084*	
H6C	0.036455	0.546043	0.799809	0.084*	
N11	0.4689 (4)	0.2402 (3)	0.56727 (14)	0.0364 (6)	
C11	0.5070 (5)	0.1972 (3)	0.50933 (17)	0.0361 (7)	
C12	0.3836 (5)	0.1269 (3)	0.46968 (18)	0.0398 (8)	
N12	0.2297 (5)	0.0979 (3)	0.48809 (17)	0.0483 (8)	
C13	0.1962 (5)	0.1411 (4)	0.5464 (2)	0.0475 (9)	
H13	0.090245	0.122120	0.560718	0.057*	
C14	0.3124 (5)	0.2120 (3)	0.58549 (19)	0.0419 (8)	
H14	0.282776	0.241054	0.625020	0.050*	
C15	0.6814 (6)	0.2263 (4)	0.4894 (2)	0.0531 (10)	
H15A	0.666872	0.245297	0.443174	0.080*	
H15B	0.758338	0.159609	0.497679	0.080*	
H15C	0.731196	0.292773	0.514520	0.080*	
C16	0.4179 (6)	0.0813 (4)	0.4037 (2)	0.0536 (11)	
H16A	0.532148	0.045737	0.408124	0.080*	
H16B	0.412715	0.145589	0.372821	0.080*	
H16C	0.330658	0.023588	0.387850	0.080*	

### Bis(2,3-dimethylpyrazine-κN)diiodidocadmium(II) (1) Atomic displacement parameters (Å^2^)

**Table:**

	*U*^11^	*U*^22^	*U*^33^	*U*^12^	*U*^13^	*U*^23^
Cd1	0.03579 (15)	0.04114 (15)	0.03721 (15)	0.00477 (10)	0.00448 (11)	0.00103 (10)
I1	0.04418 (16)	0.05245 (16)	0.05978 (19)	0.01851 (11)	0.00745 (12)	0.00847 (12)
I2	0.05478 (19)	0.05355 (18)	0.0691 (2)	−0.00347 (12)	−0.00479 (15)	0.02270 (14)
N1	0.0339 (15)	0.0374 (14)	0.0308 (15)	0.0023 (11)	0.0032 (12)	−0.0016 (11)
C1	0.0365 (18)	0.0338 (16)	0.0343 (18)	0.0034 (13)	0.0044 (15)	−0.0041 (13)
C2	0.0377 (18)	0.0375 (17)	0.0361 (19)	0.0008 (14)	0.0022 (15)	−0.0045 (14)
N2	0.0422 (18)	0.0588 (19)	0.0328 (17)	−0.0024 (14)	0.0058 (14)	−0.0035 (14)
C3	0.046 (2)	0.060 (2)	0.0289 (18)	0.0045 (18)	0.0005 (16)	0.0044 (16)
C4	0.038 (2)	0.050 (2)	0.0359 (19)	0.0062 (16)	−0.0010 (16)	0.0008 (16)
C5	0.054 (3)	0.077 (3)	0.045 (2)	0.029 (2)	0.013 (2)	0.015 (2)
C6	0.052 (3)	0.066 (3)	0.053 (3)	0.019 (2)	0.016 (2)	−0.003 (2)
N11	0.0345 (15)	0.0451 (15)	0.0293 (15)	0.0042 (12)	0.0033 (12)	0.0009 (12)
C11	0.0341 (18)	0.0445 (18)	0.0298 (17)	0.0089 (14)	0.0050 (14)	0.0055 (14)
C12	0.042 (2)	0.0453 (19)	0.0303 (17)	0.0128 (15)	−0.0006 (15)	0.0045 (14)
N12	0.0419 (19)	0.063 (2)	0.0390 (18)	0.0025 (15)	0.0012 (15)	−0.0026 (15)
C13	0.034 (2)	0.065 (2)	0.044 (2)	0.0023 (17)	0.0066 (17)	0.0017 (18)
C14	0.039 (2)	0.051 (2)	0.0365 (19)	0.0053 (16)	0.0091 (16)	−0.0019 (16)
C15	0.046 (2)	0.070 (3)	0.046 (2)	−0.003 (2)	0.0157 (19)	−0.006 (2)
C16	0.056 (3)	0.070 (3)	0.034 (2)	0.011 (2)	0.0022 (18)	−0.0124 (19)

### Bis(2,3-dimethylpyrazine-κN)diiodidocadmium(II) (1) Geometric parameters (Å, º)

**Table:**

Cd1—N1	2.329 (3)	C6—H6B	0.9600
Cd1—N11	2.331 (3)	C6—H6C	0.9600
Cd1—I2	2.7084 (4)	N11—C14	1.349 (5)
Cd1—I1	2.7309 (4)	N11—C11	1.350 (5)
N1—C1	1.338 (4)	C11—C12	1.405 (5)
N1—C4	1.348 (5)	C11—C15	1.494 (6)
C1—C2	1.408 (5)	C12—N12	1.334 (5)
C1—C5	1.499 (5)	C12—C16	1.503 (5)
C2—N2	1.332 (5)	N12—C13	1.346 (5)
C2—C6	1.504 (6)	C13—C14	1.372 (6)
N2—C3	1.337 (5)	C13—H13	0.9300
C3—C4	1.373 (6)	C14—H14	0.9300
C3—H3	0.9300	C15—H15A	0.9600
C4—H4	0.9300	C15—H15B	0.9600
C5—H5A	0.9600	C15—H15C	0.9600
C5—H5B	0.9600	C16—H16A	0.9600
C5—H5C	0.9600	C16—H16B	0.9600
C6—H6A	0.9600	C16—H16C	0.9600
			
N1—Cd1—N11	97.83 (10)	C2—C6—H6C	109.5
N1—Cd1—I2	113.60 (7)	H6A—C6—H6C	109.5
N11—Cd1—I2	113.12 (7)	H6B—C6—H6C	109.5
N1—Cd1—I1	102.85 (7)	C14—N11—C11	118.0 (3)
N11—Cd1—I1	106.28 (7)	C14—N11—Cd1	119.2 (2)
I2—Cd1—I1	120.373 (14)	C11—N11—Cd1	122.8 (2)
C1—N1—C4	117.6 (3)	N11—C11—C12	119.9 (3)
C1—N1—Cd1	124.9 (2)	N11—C11—C15	118.3 (3)
C4—N1—Cd1	117.3 (2)	C12—C11—C15	121.9 (3)
N1—C1—C2	120.1 (3)	N12—C12—C11	122.1 (4)
N1—C1—C5	118.9 (3)	N12—C12—C16	116.4 (4)
C2—C1—C5	121.0 (3)	C11—C12—C16	121.5 (4)
N2—C2—C1	122.0 (3)	C12—N12—C13	116.6 (3)
N2—C2—C6	116.8 (4)	N12—C13—C14	122.6 (4)
C1—C2—C6	121.2 (3)	N12—C13—H13	118.7
C2—N2—C3	116.8 (3)	C14—C13—H13	118.7
N2—C3—C4	122.2 (4)	N11—C14—C13	120.8 (4)
N2—C3—H3	118.9	N11—C14—H14	119.6
C4—C3—H3	118.9	C13—C14—H14	119.6
N1—C4—C3	121.3 (4)	C11—C15—H15A	109.5
N1—C4—H4	119.4	C11—C15—H15B	109.5
C3—C4—H4	119.4	H15A—C15—H15B	109.5
C1—C5—H5A	109.5	C11—C15—H15C	109.5
C1—C5—H5B	109.5	H15A—C15—H15C	109.5
H5A—C5—H5B	109.5	H15B—C15—H15C	109.5
C1—C5—H5C	109.5	C12—C16—H16A	109.5
H5A—C5—H5C	109.5	C12—C16—H16B	109.5
H5B—C5—H5C	109.5	H16A—C16—H16B	109.5
C2—C6—H6A	109.5	C12—C16—H16C	109.5
C2—C6—H6B	109.5	H16A—C16—H16C	109.5
H6A—C6—H6B	109.5	H16B—C16—H16C	109.5
			
C4—N1—C1—C2	−2.0 (5)	C14—N11—C11—C12	0.9 (5)
Cd1—N1—C1—C2	−178.2 (2)	Cd1—N11—C11—C12	−179.6 (2)
C4—N1—C1—C5	178.1 (4)	C14—N11—C11—C15	−179.0 (3)
Cd1—N1—C1—C5	1.9 (5)	Cd1—N11—C11—C15	0.5 (4)
N1—C1—C2—N2	1.6 (5)	N11—C11—C12—N12	−1.8 (5)
C5—C1—C2—N2	−178.5 (4)	C15—C11—C12—N12	178.1 (4)
N1—C1—C2—C6	−179.8 (4)	N11—C11—C12—C16	177.5 (3)
C5—C1—C2—C6	0.1 (6)	C15—C11—C12—C16	−2.6 (6)
C1—C2—N2—C3	−0.5 (5)	C11—C12—N12—C13	1.1 (5)
C6—C2—N2—C3	−179.1 (4)	C16—C12—N12—C13	−178.2 (4)
C2—N2—C3—C4	−0.2 (6)	C12—N12—C13—C14	0.3 (6)
C1—N1—C4—C3	1.3 (5)	C11—N11—C14—C13	0.5 (5)
Cd1—N1—C4—C3	177.8 (3)	Cd1—N11—C14—C13	−179.0 (3)
N2—C3—C4—N1	−0.3 (6)	N12—C13—C14—N11	−1.2 (6)

### Bis(2,3-dimethylpyrazine-κN)diiodidocadmium(II) (1) Hydrogen-bond geometry (Å, º)

**Table:**

*D*—H···*A*	*D*—H	H···*A*	*D*···*A*	*D*—H···*A*
C4—H4···I1	0.93	3.13	3.848 (4)	136
C5—H5*A*···I2^i^	0.96	3.26	4.151 (5)	156
C15—H15*C*···I2	0.96	3.28	4.186 (5)	158

Symmetry code: (i) *x*−1, *y*, *z*.

### catena-Poly[[diiodidocadmium(II)]-µ-2,3-dimethylpyrazine-κ2N1:N4] (2) Crystal data

**Table:**

[CdI_2_(C_6_H_8_N_2_)]	*D*_x_ = 2.846 Mg m^−^^3^
*M**_r_* = 474.34	Mo *K*α radiation, λ = 0.71073 Å
Orthorhombic, *P**b**c**m*	Cell parameters from 120 reflections
*a* = 8.639 (1) Å	θ = 10.0–15.0°
*b* = 12.183 (1) Å	µ = 7.50 mm^−^^1^
*c* = 10.519 (1) Å	*T* = 293 K
*V* = 1107.11 (19) Å^3^	Block, colorless
*Z* = 4	0.12 × 0.10 × 0.08 mm
*F*(000) = 848	

### catena-Poly[[diiodidocadmium(II)]-µ-2,3-dimethylpyrazine-κ2N1:N4] (2) Data collection

**Table:**

Stoe Stadi-4 diffractometer	1022 reflections with *I* > 2σ(*I*)
Phi scans	*R*_int_ = 0.018
Absorption correction: ψ scan (REDU; Stoe 1990)	θ_max_ = 27.0°, θ_min_ = 2.4°
*T*_min_ = 0.300, *T*_max_ = 0.372	*h* = −11→1
1623 measured reflections	*k* = −1→15
1273 independent reflections	*l* = −13→1

### catena-Poly[[diiodidocadmium(II)]-µ-2,3-dimethylpyrazine-κ2N1:N4] (2) Refinement

**Table:**

Refinement on *F*^2^	Hydrogen site location: inferred from neighbouring sites
Least-squares matrix: full	H-atom parameters constrained
*R*[*F*^2^ > 2σ(*F*^2^)] = 0.023	*w* = 1/[σ^2^(*F*_o_^2^) + (0.0229*P*)^2^ + 1.2591*P*] where *P* = (*F*_o_^2^ + 2*F*_c_^2^)/3
*wR*(*F*^2^) = 0.056	(Δ/σ)_max_ = 0.001
*S* = 1.04	Δρ_max_ = 0.65 e Å^−^^3^
1273 reflections	Δρ_min_ = −0.61 e Å^−^^3^
57 parameters	Extinction correction: SHELXL-2016/6 (Sheldrick 2015b), Fc^*^=kFc[1+0.001xFc^2^λ^3^/sin(2θ)]^-1/4^
0 restraints	Extinction coefficient: 0.00184 (13)
Primary atom site location: dual	

### catena-Poly[[diiodidocadmium(II)]-µ-2,3-dimethylpyrazine-κ2N1:N4] (2) Special details

**Table:**

Geometry. All esds (except the esd in the dihedral angle between two l.s. planes) are estimated using the full covariance matrix. The cell esds are taken into account individually in the estimation of esds in distances, angles and torsion angles; correlations between esds in cell parameters are only used when they are defined by crystal symmetry. An approximate (isotropic) treatment of cell esds is used for estimating esds involving l.s. planes.

### catena-Poly[[diiodidocadmium(II)]-µ-2,3-dimethylpyrazine-κ2N1:N4] (2) Fractional atomic coordinates and isotropic or equivalent isotropic displacement parameters (Å^2^)

**Table:**

	*x*	*y*	*z*	*U*_iso_*/*U*_eq_	
Cd1	0.40619 (5)	0.46580 (4)	0.250000	0.03304 (13)	
I1	0.71252 (5)	0.51634 (4)	0.250000	0.04672 (14)	
I2	0.20422 (5)	0.63429 (4)	0.250000	0.05234 (16)	
N1	0.3756 (4)	0.3358 (3)	0.4137 (3)	0.0340 (7)	
C1	0.2422 (5)	0.2952 (3)	0.4580 (4)	0.0324 (9)	
C2	0.5081 (5)	0.2913 (3)	0.4560 (5)	0.0422 (10)	
H2	0.601953	0.317212	0.424685	0.051*	
C3	0.0932 (5)	0.3485 (4)	0.4212 (5)	0.0508 (12)	
H3A	0.114314	0.415217	0.375865	0.076*	
H3B	0.034355	0.364828	0.496250	0.076*	
H3C	0.035105	0.299658	0.367747	0.076*	

### catena-Poly[[diiodidocadmium(II)]-µ-2,3-dimethylpyrazine-κ2N1:N4] (2) Atomic displacement parameters (Å^2^)

**Table:**

	*U*^11^	*U*^22^	*U*^33^	*U*^12^	*U*^13^	*U*^23^
Cd1	0.0352 (2)	0.0294 (2)	0.0346 (2)	−0.00002 (17)	0.000	0.000
I1	0.0378 (2)	0.0481 (3)	0.0543 (3)	−0.01019 (19)	0.000	0.000
I2	0.0471 (3)	0.0339 (2)	0.0760 (4)	0.00816 (18)	0.000	0.000
N1	0.0378 (17)	0.0334 (17)	0.0306 (17)	−0.0009 (15)	0.0023 (15)	0.0022 (15)
C1	0.037 (2)	0.036 (2)	0.025 (2)	−0.0011 (17)	−0.0039 (16)	−0.0021 (18)
C2	0.036 (2)	0.040 (2)	0.051 (3)	0.0012 (18)	0.000 (2)	0.005 (2)
C3	0.041 (2)	0.061 (3)	0.050 (3)	0.000 (2)	0.001 (2)	0.023 (3)

### catena-Poly[[diiodidocadmium(II)]-µ-2,3-dimethylpyrazine-κ2N1:N4] (2) Geometric parameters (Å, º)

**Table:**

Cd1—N1^i^	2.354 (3)	C1—C3	1.493 (6)
Cd1—N1	2.354 (3)	C2—C2^ii^	1.368 (9)
Cd1—I2	2.6941 (6)	C2—H2	0.9300
Cd1—I1	2.7170 (7)	C3—H3A	0.9600
N1—C1	1.338 (5)	C3—H3B	0.9600
N1—C2	1.343 (5)	C3—H3C	0.9600
C1—C1^ii^	1.412 (8)		
			
N1^i^—Cd1—N1	94.00 (17)	C1^ii^—C1—C3	120.1 (2)
N1^i^—Cd1—I2	116.09 (8)	N1—C2—C2^ii^	121.4 (2)
N1—Cd1—I2	116.09 (8)	N1—C2—H2	119.3
N1^i^—Cd1—I1	105.18 (8)	C2^ii^—C2—H2	119.3
N1—Cd1—I1	105.18 (8)	C1—C3—H3A	109.5
I2—Cd1—I1	117.27 (2)	C1—C3—H3B	109.5
C1—N1—C2	118.0 (4)	H3A—C3—H3B	109.5
C1—N1—Cd1	126.9 (3)	C1—C3—H3C	109.5
C2—N1—Cd1	114.7 (3)	H3A—C3—H3C	109.5
N1—C1—C1^ii^	120.4 (2)	H3B—C3—H3C	109.5
N1—C1—C3	119.4 (4)		
			
C2—N1—C1—C1^ii^	−3.0 (7)	Cd1—N1—C1—C3	−12.3 (6)
Cd1—N1—C1—C1^ii^	169.6 (4)	C1—N1—C2—C2^ii^	−1.6 (8)
C2—N1—C1—C3	175.1 (4)	Cd1—N1—C2—C2^ii^	−175.0 (5)

Symmetry codes: (i) *x*, *y*, −*z*+1/2; (ii) *x*, −*y*+1/2, −*z*+1.

### catena-Poly[[diiodidocadmium(II)]-µ-2,3-dimethylpyrazine-κ2N1:N4] (2) Hydrogen-bond geometry (Å, º)

**Table:**

*D*—H···*A*	*D*—H	H···*A*	*D*···*A*	*D*—H···*A*
C2—H2···I1	0.93	3.19	3.915 (5)	136
C3—H3*A*···I2	0.96	3.08	4.036 (5)	175
C3—H3*C*···I2^iii^	0.96	3.14	4.081 (5)	167

Symmetry code: (iii) −*x*, *y*−1/2, −*z*+1/2.
